# Short-type single-balloon enteroscope-assisted endoscopic retrograde cholangiopancreatography for complete occlusion of the biliary-enteric anastomosis

**DOI:** 10.1055/a-2767-9990

**Published:** 2026-02-04

**Authors:** Yi-Fan Qu, Li Wang, Jing-Zheng Liu, Shao-Bin Luo, Quan-Lin Li, Ping-Hong Zhou, Wei-Feng Chen

**Affiliations:** 1 92323 Endoscopy Center and Endoscopy Research Institute, Zhongshan Hospital, Fudan University, Shanghai, China; 2 Endoscopy Center, Shanghai Geriatric Medical Center, Shanghai, China; 3 Shanghai Collaborative Innovation Center of Endoscopy, Shanghai, China

A 33-year-old woman had previously undergone laparoscopic excision of a congenital choledochal cyst and Roux-en-Y choledochojejunostomy. Four months later, she developed recurrent fever and jaundice and underwent percutaneous cholangioscopy, but the procedure failed to access the obstructed bile duct. Then, percutaneous transhepatic cholangiodrainage (PTCD) was performed, and magnetic resonance cholangiopancreatography revealed a significant anastomotic stricture.

**Fig. 1:**
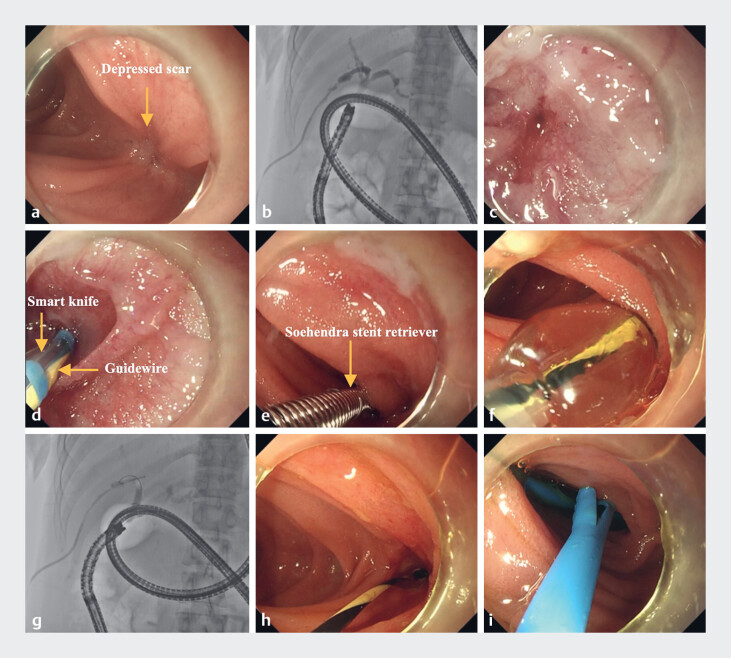
**a** A depressed scar identified on the jejunal wall near the hepatic hilum. **b** PTCD contrast injection demonstrating the complete occlusion of the anastomosis. **c** and **d** An endoscopic view confirming the complete occlusion of the anastomosis, and the smart knife tip assisted guidewire punctured the scar. **e** A stent retriever was successfully advanced over the guidewire to traverse and dilate the stricture. **f** and **g** Balloon dilation of the stricture, and X-ray fluoroscopy showed successful balloon dilation. **h** The endoscopy showed successful balloon dilation. **i** Plastic stents placed in the right and left hepatic ducts. PTCD, percutaneous transhepatic cholangiodrainage.

A short-type single-balloon enteroscope with a transparent cap and a balloon overtube was proceeded smoothly to the blind end of the afferent loop ( Video 1 ). Under X-ray fluoroscopic guidance, a scar depression was seen in the jejunal wall near the hepatic hilum ( Fig. 1 **a** ), which was revealed as a completely occluded anastomosis by PTCD tube angiography ( Fig. 1 **b** ). After repeated failed guidewire puncture attempts, a Smart knife tip was pressed against the scar depression, combined with a guidewire puncture of the scar, allowing the guidewire to eventually enter the bile duct ( Fig. 1 **c, d** ). Conventional balloon dilation could not pass the stricture, so a stent retriever was used through repeated drilling and advancement over the guidewire, ultimately successfully dilating the tract ( Fig. 1 **e** ). This was followed by 6 mm balloon dilation and successful dilation confirmed by fluoroscopy ( Fig. 1 **f, g** ), with endoscopic confirmation of good dilation results ( Fig. 1 **h** ). Finally, two plastic stents (8.5 Fr × 7 cm and 7 Fr × 7 cm) were placed in the right and left hepatic ducts ( Fig. 1 **i** ). The postoperative PTCD volume decreased significantly, and the bilirubin and liver enzymes decreased markedly. The patient was discharged uneventfully on postoperative day 2. At 2-month follow-up, the patient was asymptomatic.

Smart knife-assisted guidewire puncture combined with dilation enabling recanalization and bilateral stenting of a completely occluded choledochojejunal anastomosis under single-balloon enteroscope and fluoroscopic guidance.Video 1

In our case, the bile duct jejunostomy was completely occluded, making guidewire puncture very difficult, and common dilators could not pass through the lumen of the guidewire puncture. This case demonstrates that smart knife-assisted guidewire puncture combined with stent retriever expansion can safely and effectively puncture and dilate strictures, restoring anastomotic patency.

Endoscopy_UCTN_Code_TTT_1AR_2AJ

